# Incident Heart Failure in Patients With Coronary Artery Disease Undergoing Percutaneous Coronary Intervention

**DOI:** 10.3389/fcvm.2021.727727

**Published:** 2021-10-04

**Authors:** Jun Gu, Zhao-fang Yin, Zuo-jun Xu, Yu-qi Fan, Chang-qian Wang, Jun-feng Zhang

**Affiliations:** Department of Cardiology, Shanghai Ninth People's Hospital, Shanghai Jiaotong University School of Medicine, Shanghai, China

**Keywords:** coronary artery disease, percutaneous coronary intervention, heart failure, prognosis, risk factor

## Abstract

**Background:** The contemporary incidence of heart failure (HF) in patients with coronary artery disease (CAD) undergoing percutaneous coronary intervention (PCI) remains unclear. This prospective cohort study was designed to study the incidence and predictors of new-onset HF in CAD patients after PCI (ChiCTR1900023033).

**Methods:** From January 2014 to December 2018, 3,910 CAD patients without HF history undergoing PCI were prospectively enrolled. Demographics, medical history, cardiovascular risk factors, cardiac parameters, and medication data were collected at baseline. Multivariable adjusted competing-risk regression analysis was performed to examine the predictors of incident HF.

**Results:** After a median follow-up of 63 months, 497 patients (12.7%) reached the primary endpoint of new-onset HF, of which 179, 110, and 208 patients (36.0, 22.1, and 41.9%) were diagnosed as having HF with reduced ejection fraction (EF) (HFrEF), HF with mid-range EF (HFmrEF), and HF with preserved EF (HFpEF), respectively. Higher B-type natriuretic peptide (BNP) or E/e′ level, lower estimated glomerular filtration rate (eGFR) level, and atrial fibrillation were the independent risk factors of new-onset HF. Gender (male) and angiotensin-converting enzyme inhibitor/angiotensin II receptor blocker (ACEI/ARB) prescription were the negative predictors of new-onset HF. Moreover, it was indicated that long-term ACEI/ARB therapy, instead of beta-blocker use, was linked to lower risks of development of all three HF subtypes (HFrEF, HFmrEF and HFpEF).

**Conclusions:** This prospective longitudinal cohort study shows that the predominant subtype of HF after PCI is HFpEF and ACEI/ARB therapy is accompanied with reduced risks of incident HF across three subtypes.

## Introduction

Coronary artery disease (CAD) is still the leading global cause of mortality (1), and patients with CAD are at higher risk for adverse cardiovascular events, including recurrent myocardial infarction (MI), arrhythmia, heart failure (HF), and stroke (2). HF may be caused by acute loss of myocardial tissue due to MI, as well as by left ventricular remodeling or severe chronic ischemia. The development of HF is particularly severe since compared to other CAD patients or MI survivors without HF, patients with HF have a several-fold increased risk of death (2, 3). Prevention and management of HF remains a major public health concern due to its enormous financial and societal burden, with an estimated annual cost of $40 billion that is predicted to increase to almost $69.7 billion by 2030 (4). Therefore, efforts to prevent the development of HF or identify high-risk patients are of great significance to individual patients and the public health community.

HF is classified into the three subgroups based on the left ventricular ejection fraction (LVEF): HF with reduced EF (HFrEF) (LVEF < 40%), HF with mid-range EF (HFmrEF) (40% ≤ LVEF < 50%), and HF with preserved EF (HFpEF) (LVEF ≥ 50%) (2). To date, there are insufficient data on the incidence of HF in CAD patients undergoing percutaneous coronary intervention (PCI). Therefore, we aimed to study the incidence and profile of HF and their predictors in a contemporary population of CAD patients receiving PCI included in our prospective longitudinal cohort registry (ChiCTR1900023033).

## Methods

### Study Population

In this prospective longitudinal cohort, we enrolled subjects with symptomatic CAD who received PCI from January 2014 to December 2018 at Shanghai Ninth People's Hospital, Shanghai Jiaotong University School of Medicine. The diagnosis of CAD included positive stress test, history of angina with ischemic change on electrocardiogram, MI attack, or angina with obvious stenosis lesion in coronary computed tomography angiography (CCTA). Symptomatic patients who received PCI either with coronary stenting or with balloon angioplasty were eligible for enrollment. Inclusion criteria were LVEF ≥ 50% and without HF previously or at baseline. Exclusion criteria were defined as end-stage renal failure [estimated glomerular filtration rate (eGFR) <30 ml/min/1.73 m^2^]; hypertrophic cardiomyopathy or infiltrative cardiomyopathy; valvular heart disease; and any serious non-cardiovascular disease with a life expectancy of 6 months or less. All procedures were conducted under the guidance of the Declaration of Helsinki and were approved by the local Ethics Committee and Independent Review Board (SH9H-2019-T160-2).

### Baseline Characteristics and Biochemical Data

Coronary angiography and revascularization procedures were conducted using standard techniques. Revascularization procedures, such as thrombectomy, pre-dilatation, stenting, and/or post-dilatation, were performed at the discretion of each operator. Pharmacotherapeutic strategies after PCI, such as antiplatelet treatments, statins, angiotensin-converting enzyme inhibitor/angiotensin II receptor blocker (ACEI/ARB), and beta-blockers, followed the CAD guidelines. Baseline characteristics were obtained from each enrolled patient including sex, age, history of hypertension, diabetes, hyperlipidemia, smoking, and cerebral vascular disease. Furthermore, biochemical data and medications as well as echocardiographic data were also collected.

### Clinical Follow-Up and Endpoints

For the present investigation, our primary outcomes of interest were the incidence of HF and its subtypes during long-term follow-up. HFrEF, HFmrEF, and HFpEF were distinguished based on LVEF of <40, 40 to 49, and ≥50%, respectively, at or close to the time of HF episode. Symptoms of HF included shortness of breath, reduced exercise tolerance, fatigue, and/or ankle swelling. The diagnosis of new-onset HF was based on the 2016 ESC-HF guideline (2). Generally, the enrolled patients received a clinical follow-up examination every 1–3 months, and symptoms and signs of HF were evaluated at each visit. The natriuretic peptide should be determined (if necessary) to identify patients who require echocardiographic demonstration of structural and/or functional changes of the heart, as it is the prerequisite for the diagnosis of HF.

### Statistical Analysis

SPSS 22.0 (SPSS Inc., Chicago, IL, USA) and Stata 16 (StataCorp, College Station, TX, USA) were used for statistical analysis. Quantitative variables were described as arithmetic means ± standard deviations and analysis by *t*-test and one-way ANOVA test, if appropriate, while qualitative variables were described as percentages (%) and numbers, and analyzed by the two-sided chi-square test. Univariate and multivariate Cox regression analyses were performed on the relevant variables to determine the predictors of the primary endpoint of new-onset HF. All predictors with a significance of *p* < 0.10 from univariate analysis and mandatory inclusion variables considered to be important predictors of clinical endpoints were entered into the multivariate model. To counteract the competing risk of death, cumulative sub-hazard ratios (SHR) of new-onset HF were estimated by competing-risk regression using the Fine and Gray model. Freedom from new-onset HF during long-term follow-up was analyzed with Kaplan–Meier statistics (log-rank test). All values were two-tailed, and a *p*-value < 0.05 was considered statistically significant.

## Results

### Patient Characteristics

A total of 4,569 patients were undergoing coronary intervention in this prospective cohort from January 2014 to December 2018, and 659 patients were excluded due to a history of HF or current HF symptoms, missing echocardiographic data, loss to follow-up, or other exclusion criteria. Finally, 3,910 patients were included in the present analysis. The baseline characteristics of enrolled patients are presented in Table 1. The patients' mean age was 67.7 ± 11.1 years, and 68.0% of patients were male. Nearly 36.0% of patients were current or former smokers, ~32.5% had diabetes, about 35.3% had hyperlipidemia, and 70.2% had hypertension. Almost 9.7 and 26.8% of patients had a history of MI and PCI, respectively. Both blood pressure and heart rate were relatively well-controlled. Among those with available data, atrial fibrillation was present in 3.0%. The use of guideline-recommended medical therapy for CAD after PCI was relatively high. Antiplatelet treatment was prescribed in 92.1% for aspirin and 97.9% for the P2Y12 inhibitor, statin in 93.7%, ACEI/ARB in 69.7%, and beta-blockers in 61.8% of registry participants.

**Table 1:** Baseline clinical characteristics and medications.

**Parameter**	**Total ***n*** = 3,910**	**Non-HF ***n*** = 3,413**	**New-onset HF ***n*** = 497**	***P*** **-value**
**Demographic characteristics**				
Age, years	67.7 ± 11.1	67.5 ± 11.2	68.6 ± 10.7	0.035
Gender, male	2,658 (68.0)	2,353 (68.9)	305 (61.4)	0.001
BMI (kg/m^2^)	24.9 ± 5.5	24.9 ± 5.7	24.7 ± 3.3	0.428
**Cardiovascular risk factors**				
Dyslipidaemia	1,381 (35.3)	1,218 (35.7)	163 (32.8)	0.208
Hypertension	2,746 (70.2)	2,378 (69.7)	368 (74.0)	0.041
Diabetes	1,269 (32.5)	1,086 (31.8)	183 (36.8)	0.027
Smoking	1,408 (36.0)	1,213 (35.5)	195 (39.2)	0.109
**Medical history**				
History of MI	381 (9.7)	319 (9.3)	62 (12.5)	0.028
Previous PCI	1,049 (26.8)	921 (27.0)	128 (25.8)	0.563
Pervious CABG	38 (1.0)	34 (1.0)	4 (0.8)	1.000
Stroke	257 (6.6)	220 (6.4)	37 (7.4)	0.401
COPD	278 (7.1)	236 (6.9)	42 (8.5)	0.223
Atrial fibrillation	117 (3.0)	79 (2.3)	38 (7.6)	<0.001
**Cardiac parameters**				
Heart rate, bpm	76.8 ± 13.6	76.8 ± 13.7	76.5 ± 13.1	0.669
SBP, mmHg	137.3 ± 20.3	137.2 ± 20.4	138.3 ± 20.2	0.233
DBP, mmHg	77.9 ± 11.2	77.9 ± 11.2	77.7 ± 11.5	0.610
**Laboratory variables**				
eGFR (mL/min/1.73 m^2^)	67.4 ± 12.1	67.5 ± 12.0	66.2 ± 12.7	0.025
Hemoglobin (g/dL)	133.3 ± 17.2	133.2 ± 17.3	134.0 ± 16.2	0.322
BNP (pg/mL)	111.9 ± 102.6	106.0 ± 99.9	152.4 ± 111.6	<0.001
Total cholesterol	4.2 ± 1.1	4.2 ± 1.1	4.1 ± 1.1	0.246
Triglyceride (mmol/L)	1.8 ± 1.1	1.8 ± 1.1	1.8 ± 1.1	0.920
HDL-C (mmol/L)	1.0 ± 0.3	1.0 ± 0.3	1.0 ± 0.3	0.673
LDL-C (mmol/L)	2.8 ± 1.0	2.8 ± 1.0	2.7 ± 1.0	0.171
**Medications**				
Aspirin	3,602 (92.1)	3,140 (92.0)	462 (93.0)	0.460
P2Y12 inhibitor	3,826 (97.9)	3,344 (98.0)	482 (97.0)	0.152
ACEI/ARB	2,727 (69.7)	2,419 (70.9)	308 (62.0)	<0.001
Beta-blocker	2,415 (61.8)	2,101 (61.6)	304 (61.2)	0.487
CCB	1,985 (50.8)	1,737 (50.9)	248 (49.9)	0.674
Statin	3,664 (93.7)	3,203 (93.8)	461 (92.8)	0.350
Diuretic	202 (5.2)	169 (5.0)	33 (6.6)	0.112
**CAD**				
SVD	1,090 (27.9)	971 (28.5)	119 (23.9)	0.061
DVD	1,727 (44.2)	1,505 (44.1)	222 (44.7)	
TVD	1,093 (28.0)	937 (27.5)	156 (31.4)	
Stent Number	1.3 ± 0.5	1.3 ± 0.5	1.3 ± 0.6	0.927
ACS	1,602 (41.0)	1,375 (40.3)	227 (45.7)	0.023
**Echo data**				
LVEF (%)	60.5 ± 4.9	60.6 ± 4.9	59.7 ± 5.0	<0.001
LAD (mm)	38.1 ± 3.6	38.0 ± 3.7	38.7 ± 3.5	<0.001
E/e'	9.7 ± 2.2	9.6 ± 2.2	10.1 ± 2.3	<0.001

*Data are expressed as mean ± SD, or n (%)*.

*BMI, body mass index; MI, myocardial infarction; PCI, percutaneous coronary intervention; CABG, coronary artery bypass graft; COPD, chronic obstructive pulmonary disease; SBP, systolic blood pressure; DBP, diastolic blood pressure; eGFR, estimated glomerular filtration rate; BNP, B-type natriuretic peptide; HDL-C, high-density lipoprotein cholesterol; LDL-C, low-density lipoprotein cholesterol; ACEI/ARB, angiotensin-converting enzyme inhibitor/angiotensin II receptor blocker; CCB, calcium channel blocker; CAD, coronary artery disease; SVD, single vessel disease; DVD, double vessel disease; TVD, triple vessel disease; ACS, acute coronary syndrome; LVEF, left ventricular ejection fraction; LAD, left atrium diameter; E/e', mitral Doppler early velocity/mitral annular early velocity*.

### Clinical Outcomes

During a median follow-up of 63 (range, 39–86) months, 497 patients (12.7%) reached the primary outcome of new-onset HF. There were substantial differences between those with and without the primary endpoint (Table 1). Patients with new-onset HF were older and more likely to be female than those without new-onset HF. Additionally, the percentage of patients with diabetes mellitus, hypertension, or atrial fibrillation was higher in the new-onset HF group. Laboratory examination revealed that the level of B-type natriuretic peptide (BNP) was significantly higher in the new-onset HF group. Estimated glomerular filtration rates (eGFRs) were significantly lower in the new-onset HF group. The proportion of acute coronary syndrome (ACS) was higher in the new-onset HF group, and ACEI/ARB prescription was more common in the non-HF group. As for the echocardiographic data, the LVEF, LAD, and E/e′ were significantly deteriorated in the new-onset HF group.

### Factors Predicting New-Onset HF

**Figure 1:**
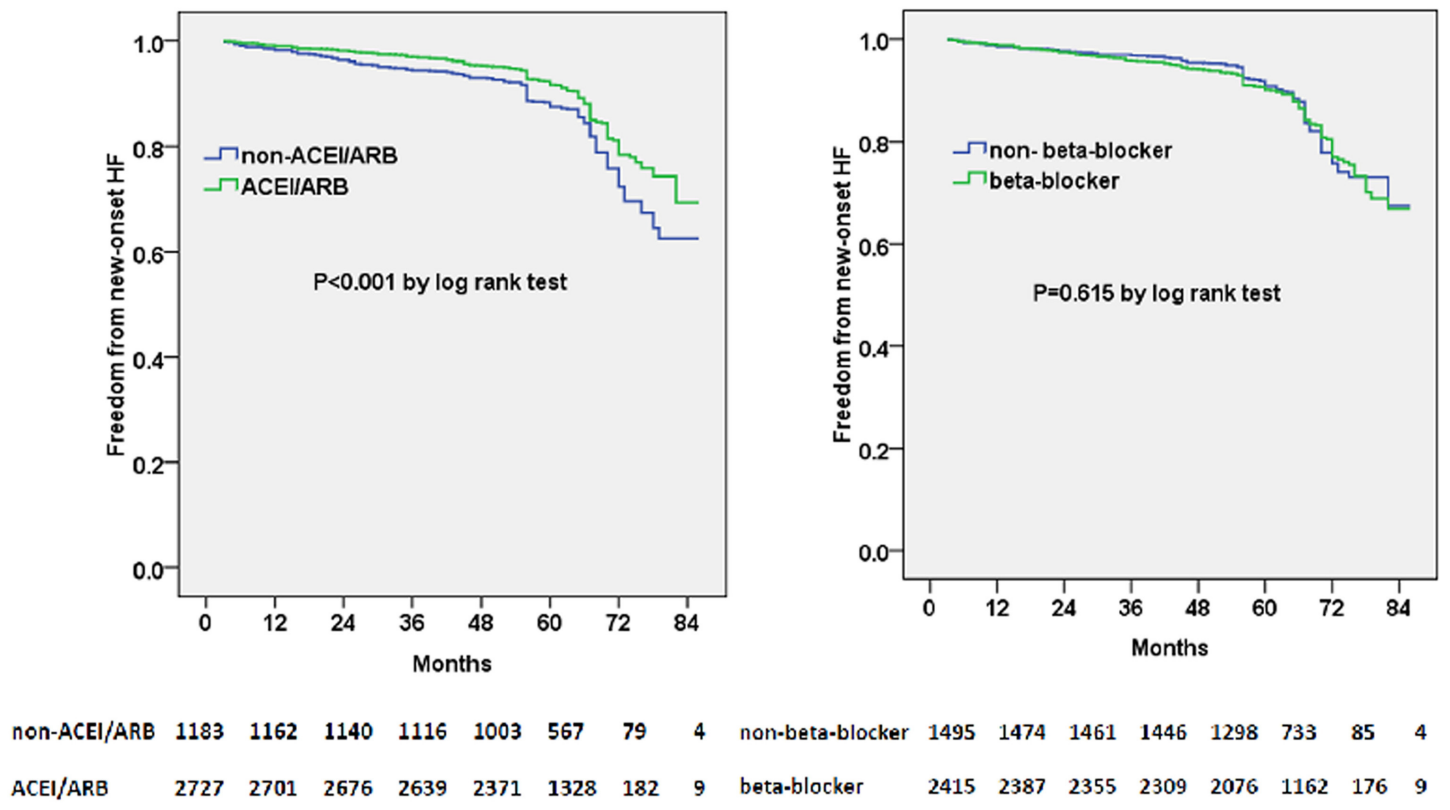
Kaplan–Meier curves of freedom from new-onset HF for different medical treatment. The numbers at the bottom of the figure are “number at risk”.

We subsequently examined the predictors of new-onset HF using multivariable adjusted competing-risk regression analysis. The death was considered as the competing risk, and 165 enrolled patients (4.2%) died during the follow-up. Table 2 shows the predictors of the primary outcome of new-onset HF. Higher BNP or E/e′ level, lower eGFR level, and atrial fibrillation were the most robust risk factors of new-onset HF. Gender (male) and ACEI/ARB prescription were negative predictors of new-onset HF. Besides, subjects prescribed with ACEI/ARB showed a reduced possibility of new-onset HF in the Kaplan–Meier plot (log-rank test, *p* < 0.001, Figure 1); however, beta-blocker prescription did not result in a reduced risk of new-onset HF (log-rank test, *p* = 0.615, Figure 1).

**Table 2:** Multivariate analysis showing predictors of new-onset HF.

	**SHR**	**95% CI**	***P*** **-value**
Age	0.991	0.982–1.007	0.135
Gender (male)	0.792	0.648–0.968	0.022
BNP	1.782	1.567–2.026	<0.001
eGFR	0.991	0.983–0.999	0.028
Previous MI	1.263	0.953–1.676	0.104
AF	3.034	2.111–4.359	0.006
Hypertension	1.129	0.919–1.387	0.247
Diabetes	1.178	0.977–1.421	0.085
ACS	1.179	0.981–1.418	0.080
ACEI/ARB	0.774	0.644–0.930	0.006
Beta-blocker	1.041	0.866–1.252	0.666
Multivessel CAD	1.074	0.956–1.207	0.228
LVEF	0.985	0.968–1.003	0.100
LAD	1.020	0.996–1.045	0.100
E/e'	1.065	1.024–1.108	0.002

*BNP, B-type natriuretic peptide; eGFR, estimated glomerular filtration rate; MI, myocardial infarction; AF, atrial fibrillation; ACS, acute coronary syndrome; ACEI/ARB, angiotensin-converting enzyme inhibitor/angiotensin II receptor blocker; CAD, coronary artery disease; LVEF, left ventricular ejection fraction; LAD, left atrium diameter; E/e', mitral Doppler early velocity/mitral annular early velocity*.

**Figure 2:**
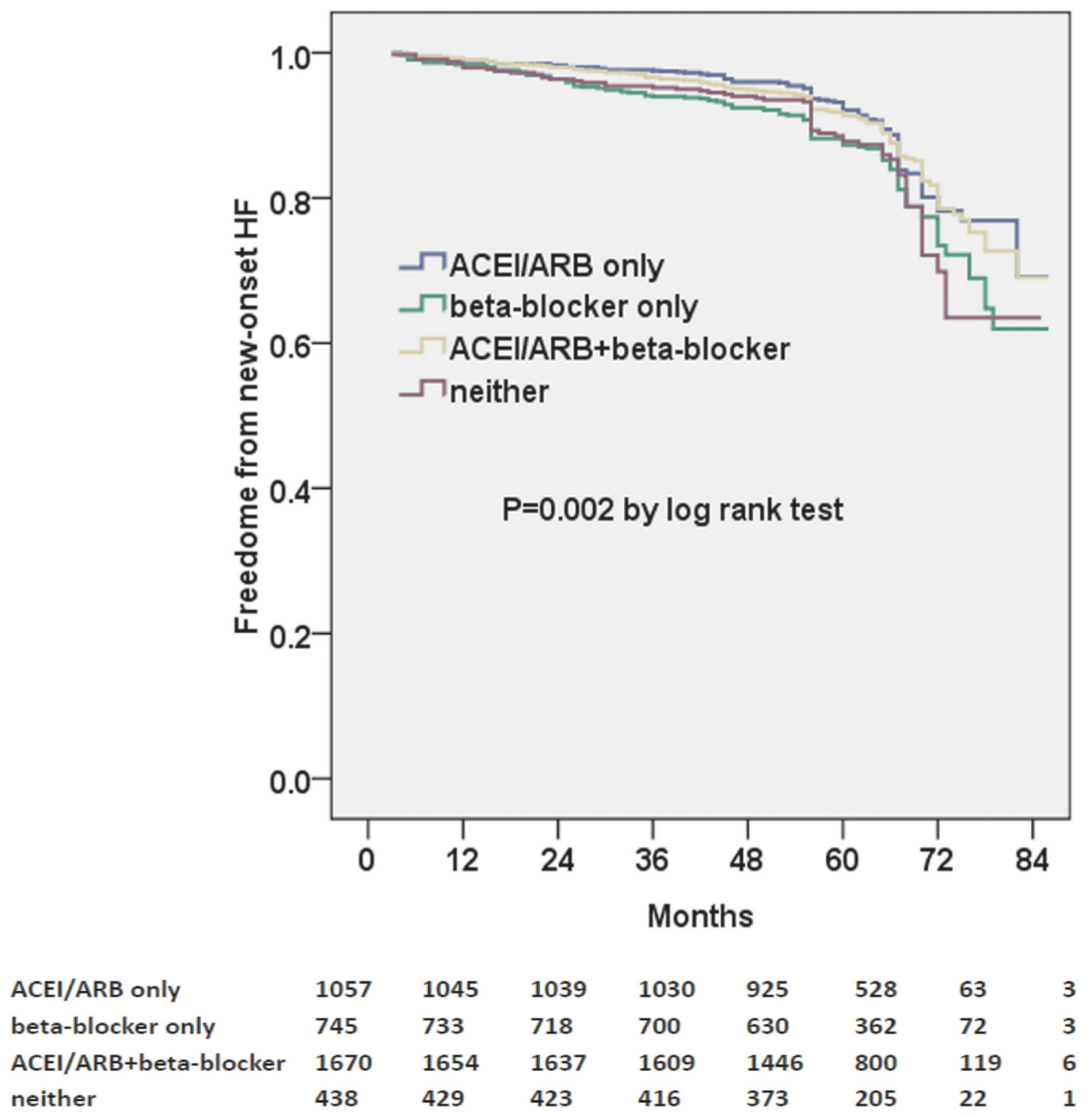
Kaplan–Meier curves of freedom from new-onset HF for different medical treatments. The numbers at the bottom of the figure are “number at risk”.

ACEI/ARB and/or beta-blocker therapy were further divided into four groups: ACEI/ARB only, beta-blocker only, ACEI/ARB+beta-blocker, and neither. Moreover, we found that ACEI/ARB only or ACEi/ARB+beta-blocker could markedly decrease the risk of new-onset HF in the Kaplan–Meier plot (log-rank test, *p* = 0.002, Figure 2). However, there was no significant difference between ACEI/ARB only and ACEI/ARB+beta-blocker with regard to the new-onset HF (11.1 vs. 11.5%, *p* = 0.731).

### Classification by LVEF Due to New Onset of HF

For 497 patients with new-onset HF, we analyzed the LVEF at or in close proximity to the time of HF episode. Consequently, 179, 110, and 208 patients (36.0, 22.1, and 41.9%) were classified into the HFrEF, HFmrEF, and HFpEF subgroups, respectively. Clinical characteristics, such as age, gender, and medical history, were comparable among the three subgroups (Supplementary Table 1). The BNP level was significantly higher and LVEF was markedly lower in the HFrEF group. The prescription rates of ACEI/ARB, beta-blocker, and diuretic were similar for all three subgroups (Supplementary Table 1).

### Predictors of Different Subtypes of New-Onset HF

**Figure 3:**
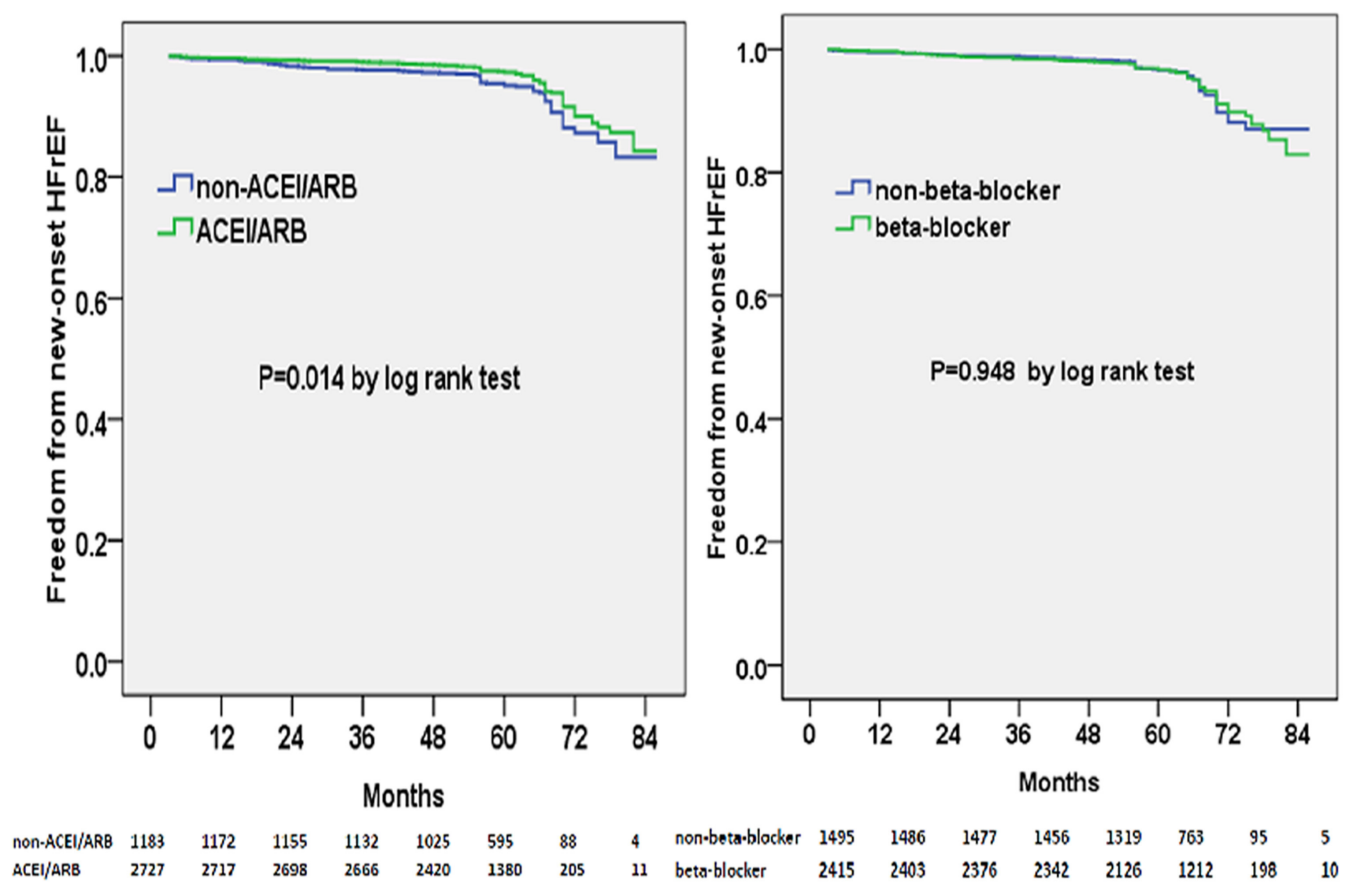
Kaplan–Meier curves of freedom from new-onset HFrEF for different medical treatments. The numbers at the bottom of the figure are “number at risk”.

**Figure 4:**
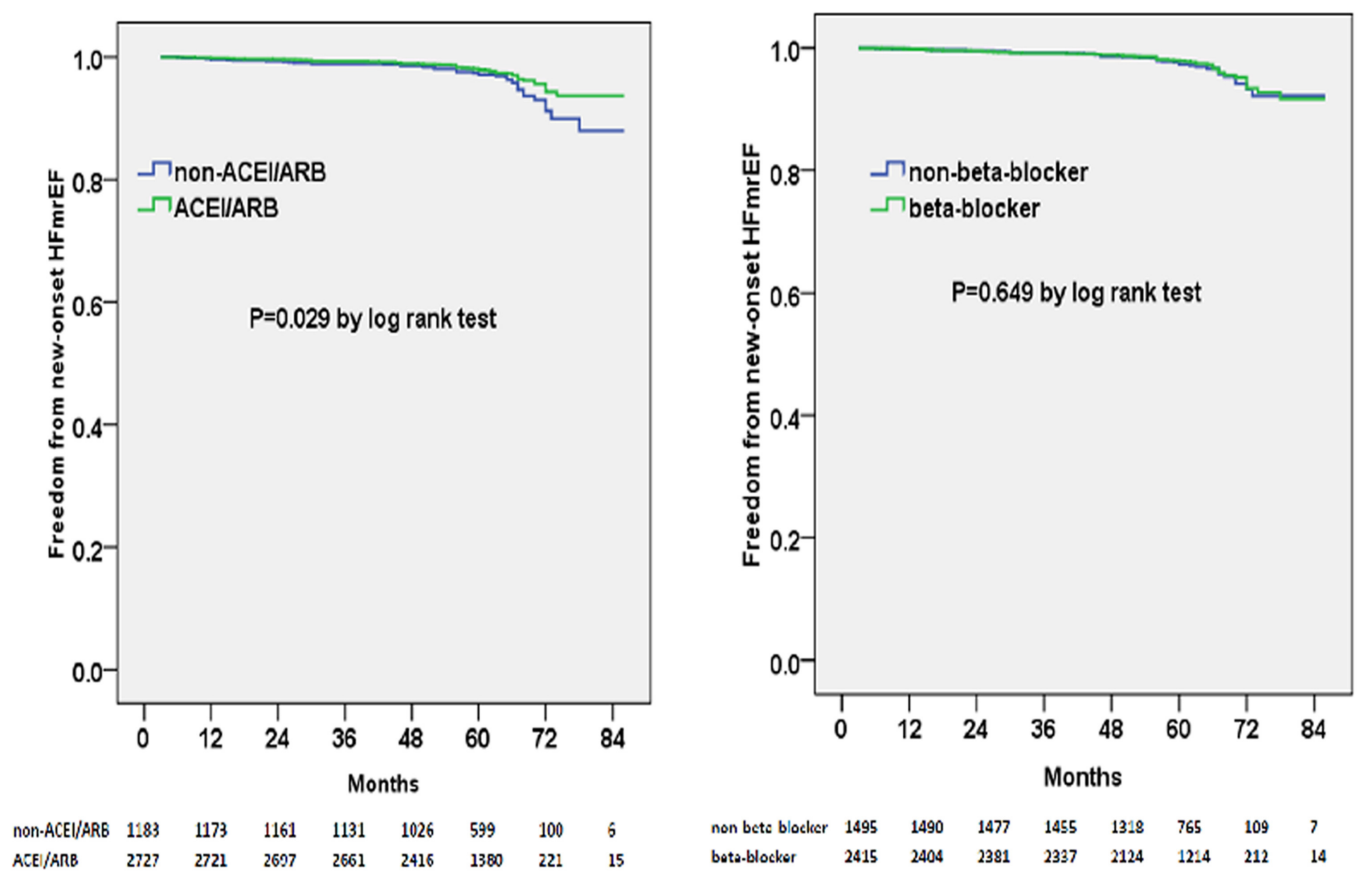
Kaplan–Meier curves of freedom from new-onset HFmrEF for different medical treatments. The numbers at the bottom of the figure are “number at risk”.

**Figure 5:**
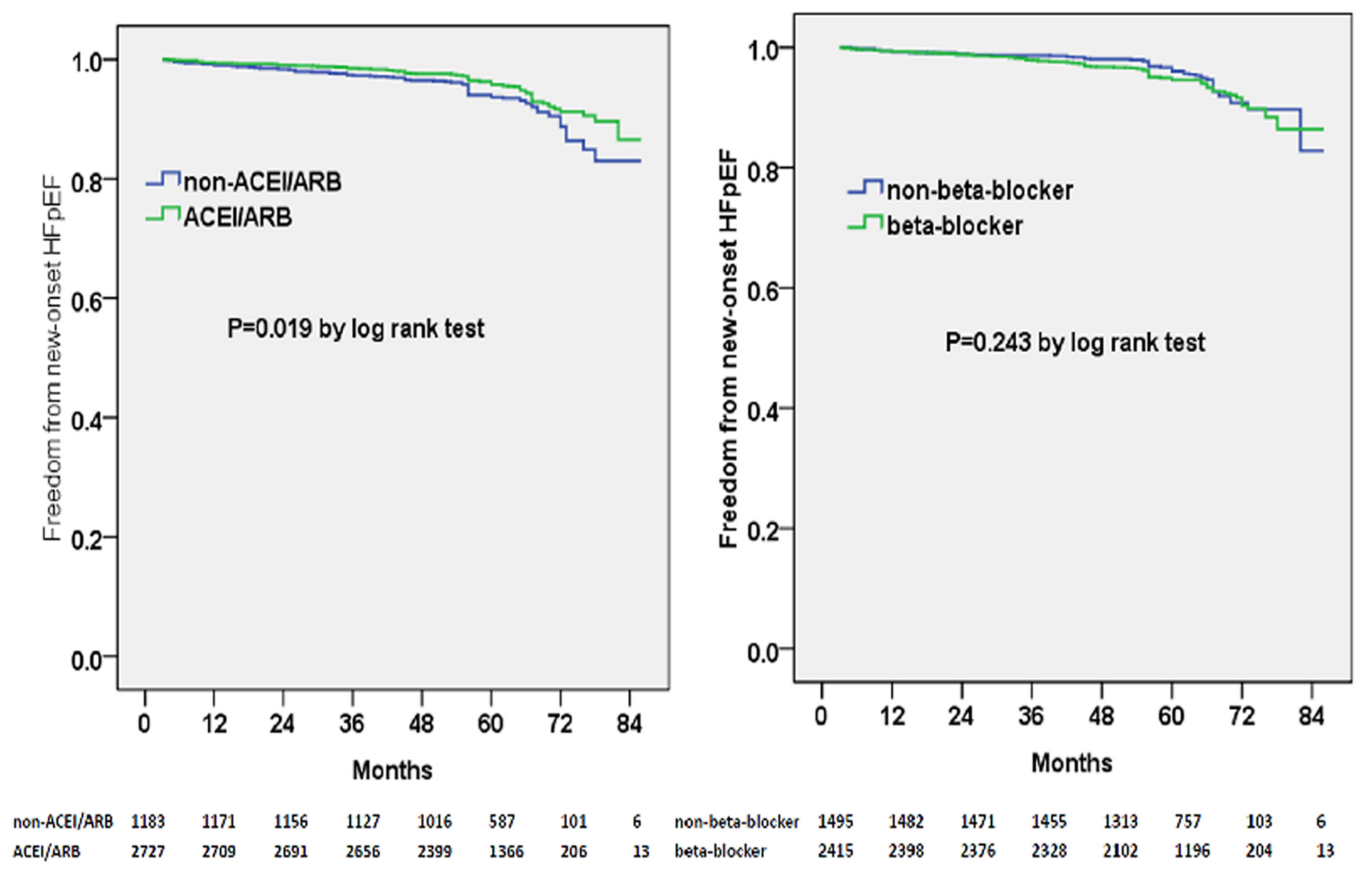
Kaplan–Meier curves of freedom from new-onset HFpEF for different medical treatments. The numbers at the bottom of the figure are “number at risk”.

Multivariable adjusted competing-risk regression analysis also revealed the risk or protective factors for the new-onset HFrEF, HFmrEF, and HFpEF, respectively (Supplementary Tables 2–4), which indicated that ACEI/ARB use, rather than beta-blocker prescription, was associated with a lower risk of HF development across the three subtypes. In survival analysis, ACEI/ARB prescription was linked to a markedly lower risk of new-onset HFrEF, HFmrEF, and HFpEF, but beta-blocker use did not appear to benefit the development of the three HF subtypes (Figures 3–5).

## Discussion

In the present study of CAD patients undergoing PCI, free from HF at baseline or previously, after a median follow-up of 63 months ~1 in eight patients achieved the primary outcome of new-onset HF. A number of predictors of new-onset HF were identified. Higher BNP or E/e′ level, lower eGFR level, and atrial fibrillation were shown to predict new-onset HF, while gender (male) and long-term ACEI/ARB prescription appeared to show lower risks for HF development. We also classified the new-onset HF patients into three subtypes (HFrEF, HFmrEF, and HFpEF) based on the LVEF. We found that 36.0% was HFrEF, 22.1% was HFmrEF, and 41.9% was HFpEF, and the frequency of post-PCI HFpEF was higher than speculated. Moreover, for the subgroup analysis, ACEI/ARB therapy was associated with lower risks of development of all three HF subtypes during the long-term follow-up.

Previous studies indicated that gender (female), renal dysfunction, atrial fibrillation, and E/e′ were associated with an increased risk of new-onset HF (5–9). Better control of these risk factors was beneficial to delay the occurrence and development of HF (5–9). Our previous studies also showed the preventive effects of ACEI/ARB for the new-onset HF (8, 9). The time of HF episodes after PCI might be related to comorbidities, myocardial remodeling, coronary lesions, and other factors. In the present study, there was a sharp increase in HF events after 4 years. Another study indicated that cardiovascular events (hospitalization for HF or new-onset HF) increased significantly after 5 years in chronic CAD (10).

In recent years, many predictive models for the development of HF have focused on patients with hypertension, MI survivors, or higher-risk CAD (11–14). As for low-risk CAD patients in the PEACE study, 12 characteristics were related to the increased risk of HF, such as older age, history of hypertension, and diabetes (15). In patients with chronic coronary syndrome (CCS) included in the CLARIFY registry, a sizeable proportion (16.4%) develop HF during a 5-year follow-up (10). During a median follow-up of 63 months in the present study, there were 497 patients (12.7%) of 3,910 patients who had a new-onset HF event. In this CAD population with documented preserved LVEF and without HF previously or at baseline who were well-treated with contemporary therapy, there was still a risk of HF development. Therefore, the timely identification of HF may lead to timely treatment, which helps to further reduce mortality and morbidity. Coronary intervention therapy has become an indispensable method for the treatment of CAD. A better understanding of the factors contributing to the eventual development of HF among CAD patients after PCI may help develop new strategies to prevent the progression of this disease and improve quality of life and overall survival.

Currently, we also reported the frequency of the occurrence of HF subtypes after PCI, and HFpEF accounted for the largest proportion of newly diagnosed HF. Another study also indicated that the predominant subtypes of HF after AMI were HFmrEF and HFpEF, or HF with non-reduced EF (13). With the increase of population aging and the increased survival rate after MI, the prevalence of HF continues to rise, among which HFpEF has become the predominant type (16). Although the progress of pharmacologic and non-pharmacologic therapies in recent years have improved the clinical outcome of HF, they are only effective for patients with HFrEF, and there is no clear treatment for patients with HFpEF. In our previous study, we utilized the machine-learning-based clustering strategy to identify three distinct phenol groups of HFpEF that differed significantly in comorbidity burden, underlying cardiac abnormalities, and long-term prognosis (17). Long-term beta-blocker or ACEI/ARB prescription was linked to a lower risk of adverse cardiovascular events in a specific subtype of HFpEF (17). Our recent studies also indicated that identification and management of high-risk patients might be the first steps toward the ultimate goal of preventing or delaying the HFpEF progression (9, 18, 19). The pathophysiological mechanism of HFpEF secondary to ischemia is exceedingly complicated. During ischemia, the passive stiffness of myocardial fibers increases, leading to impaired myocardial relaxation, and then the left ventricular filling pressure increases, further restricting myocardial blood flow, aggravating ischemia, leading to pulmonary congestion and shortness of breath, which are the hallmarks of HF (16).

ACEI/ARB has been shown to reduce adverse cardiovascular events in patients with HF, MI combined with HF, and high-risk CAD (20–22). In the HOPE study, ramipril significantly reduced the rates of composite endpoints of death from a cardiovascular cause, MI, and stroke in high-risk patients who are not identified as a low LVEF or HF (20). In the EUROPA study, among patients with stable CAD without apparent HF, perindopril also could significantly improve outcomes (23). Moreover, the PEACE study showed that ACEI therapy significantly reduced the risk of HF in the low-risk CAD population (15). Further, a meta-analysis of HOPE, EUROPA, and PEACE studies demonstrated that ACEI therapy reduced serious vascular events in patients with atherosclerosis without known evidence of left ventricular systolic dysfunction or HF (24). In the present study, ACEI/ARB use reduced the risk of new-onset HFrEF, HFmrEF, or HFpEF in CAD patients after PCI.

In CAD patients without HF or left ventricular systolic dysfunction, the benefit of conventional beta-blocker therapy is unclear. Beta-blocker therapy did not affect 30-day major adverse cardiovascular events (MACEs) or 1-year survival after MI in patients without HF or reduced LVEF (25). However, beta-blocker treatment at discharge has been shown to be associated with a significant reduction in 1-year mortality in patients receiving PCI for unstable angina and with sufficient LVEF (26). Moreover, ambiguous results have been reported on the clinical effects of beta-blocker in acute coronary syndrome (ACS) patients without HF after successful PCI (27–30). It is generally believed that β-blockers can reduce adverse cardiac events, which to some extent supports the widespread use of β-blockers in CAD patients. American guidelines recommend that CAD patients with no contraindications receive oral beta-blocker therapy during hospitalization, which should not be suspended even after discharge, regardless of whether there is left ventricular dysfunction (class I, level of evidence B). However, the European guidelines for beta-blocker therapy for patients with sufficient LVEF are indicated as Class IIa (31–34). These recommendations are primarily derived from studies conducted in the pre-reperfusion era or studies in HF patients. However, a recent meta-analysis in the MI population revealed that beta-blockers did not reduce mortality in the reperfusion era (30). In this study, we aimed to investigate the relationship between beta-blocker therapy at discharge and long-term HF development in the CAD population who received PCI with adequate left ventricular function, and the results indicated that beta-blocker use did not significantly lower the risk of new-onset HF or the developments of HFrEF, HFmrEF, or HFpEF subtypes.

## Limitations

There are several limitations to this study. First, this was a single-center study, and the selection bias cannot completely be ruled out. Second, only Chinese patients were included in the study. Other populations were not enrolled and assessed. Third, the numbers of newly diagnosed with HFpEF, HFmrEF, and HFrEF were relatively small. Despite these limitations, this study expands our understanding of predictors of HF and HF subtypes among the CAD population after PCI and demonstrated the benefits of ACEI/ARB in reducing the HF risk across the three subtypes.

## Conclusion

This analysis shows that several traditional and easily available factors are linked to an increased risk of HF development in the CAD population after PCI. ACEI/ARB rather than the beta-blocker reduces the risk of new-onset HF across three subtypes among this population irrespective of these factors. Early identification of high-risk patients for HF development and more aggressive secondary prevention efforts may help to further reduce mortality and morbidity in this population.

## Data Availability Statement

The raw data supporting the conclusions of this article will be made available by the authors, without undue reservation.

## Ethics Statement

The studies involving human participants were reviewed and approved by the Ethics Committee and Independent Review Board of Shanghai ninth people's hospital, Shanghai Jiaotong University School of Medicine. The patients/participants provided their written informed consent to participate in this study.

## Author Contributions

JG and J-fZ designed this research. Z-fY and Z-jX collected data. Y-qF analyzed data. JG and C-qW wrote the manuscript. All authors contributed to the article and approved the submitted version.

## Funding

This study was supported by the National Nature Science Foundation of China (82070381, 81670293), Clinical Research Program (JYLJ201803), Multidisciplinary Team (201911), and Biobank for Coronary Heart Disease (YBKA201910) of Shanghai 9th People's Hospital, Natural Science Foundation of Shanghai (20ZR1431100).

## Conflict of Interest

The authors declare that the research was conducted in the absence of any commercial or financial relationships that could be construed as a potential conflict of interest.

## Publisher's Note

All claims expressed in this article are solely those of the authors and do not necessarily represent those of their affiliated organizations, or those of the publisher, the editors and the reviewers. Any product that may be evaluated in this article, or claim that may be made by its manufacturer, is not guaranteed or endorsed by the publisher.
