# Supplementary material for: Incident Heart Failure in Patients With Coronary Artery Disease Undergoing Percutaneous Coronary Intervention
Source: Front Cardiovasc Med. 2021 Oct 4;8:727727. doi: 10.3389/fcvm.2021.727727 (PMC8520925; doi:10.3389/fcvm.2021.727727)
Supplement: Supplementary file 1 [file Table_1.docx]

**Table S1 Baseline clinical characteristics and medications in three HF subtypes**

| parameter | HFrEF  n=179 | HFmrEF  n=110 | HFpEF  n=208 | P value |
| --- | --- | --- | --- | --- |
| **demographic characteristics** |  |  |  |  |
| age, years | 68.2±10.5 | 68.1±11.1 | 69.3±10.6 | 0.496 |
| gender,male | 115(64.2) | 68(61.8) | 122(58.7) | 0.527 |
| BMI (kg/m^2^) | 24.7±3.1 | 24.6±3.2 | 24.7±3.5 | 0.942 |
| **cardiovascular risk factors** |  |  |  |  |
| dyslipidaemia | 65(36.3) | 35(31.8) | 63(30.3) | 0.439 |
| hypertension | 126(70.4) | 79(71.8) | 163(78.4) | 0.170 |
| diabetes | 60(33.5) | 39(35.5) | 84(40.4) | 0.357 |
| smoking | 78(43.6) | 42(38.2) | 75(36.1) | 0.309 |
| **medical history** |  |  |  |  |
| history of MI | 28(15.6) | 13(11.8) | 21(10.1) | 0.251 |
| previous PCI | 52(29.1) | 26(23.6) | 50(24.0) | 0.450 |
| pervious CABG | 1(0.6) | 2(1.8) | 1(0.5) | 0.444 |
| stroke | 14(7.8) | 6(5.5) | 17(8.2) | 0.661 |
| COPD | 17(9.5) | 9(8.2) | 16(7.7) | 0.811 |
| atrial fibrillation | 10(5.6) | 5(4.5) | 23(11.1) | 0.050 |
| **cardiac parameters** |  |  |  |  |
| heart rate, bpm | 75.8±13.6 | 75.7±13.4 | 76.7±12.4 | 0.740 |
| SBP, mmHg | 137.3±20.8 | 138.2±16.3 | 139.3±21.4 | 0.619 |
| DBP, mmHg | 77.5 ±11.6 | 77.0±10.3 | 78.2±12.0 | 0.665 |
| **laboratory variables** |  |  |  |  |
| eGFR (mL/min/1.73 m^2^) | 67.0±12.0 | 67.3±12.8 | 65.0±13.2 | 0.194 |
| haemoglobin (g/dL) | 134.2±16.1 | 135.2±15.3 | 133.3±16.8 | 0.619 |
| BNP (pg/mL) | 166.6±103.0 | 160.5±91.0. | 136.1±126.0 | 0.019 |
| total cholesterol  (mmol/L) | 4.2±1.2 | 4.1±1.0 | 4.1±1.1 | 0.262 |
| triglyceride  (mmol/L) | 1.8±1.2 | 1.9±1.5 | 1.7±0.9 | 0.244 |
| HDL-C  (mmol/L) | 1.0±0.3 | 1.0±0.3 | 1.0±0.3 | 0.405 |
| LDL-C  (mmol/L) | 2.8±1.0 | 2.6±1.0 | 2.7±1.0 | 0.342 |
| **medications** |  |  |  |  |
| aspirin | 165(92.2) | 103(93.6) | 194(93.2) | 0.872 |
| P2Y12 inhibitors | 173(96.6) | 109(99.1) | 200(96.2) | 0.350 |
| ACEI/ARB | **110(61.5)** | **67(60.9)** | **131(63.0)** | **0.922** |
| beta-blocker | 111(62.0) | 66(60.0) | 137(65.9) | 0.532 |
| CCB | 90(50.3) | 61(55.5) | 97(46.6) | 0.327 |
| statin | 170(95.0) | 104(94.5) | 187(89.9) | 0.124 |
| diuretic | 12(6.7) | 6(5.5) | 15(7.2) | 0.870 |
| **CAD** |  |  |  |  |
| SVD | 52(29.1) | 22(20.0) | 45(21.6) | 0.310 |
| DVD | 71(39.7) | 52(47.3) | 99(47.6) |  |
| TVD | 56(31.3) | 36(32.7) | 64(30.8) |  |
| stent number | 1.3±0.5 | 1.3±0.6 | 1.3±0.6 | 0.550 |
| ACS | 73(40.8) | 52(47.3) | 102(49.0) | 0.248 |
| **echo data** |  |  |  |  |
| LVEF(%) | 58.9±4.7 | 59.6±5.2 | 60.3±5.2 | 0.024 |
| LAD (mm) | 39.0±3.6 | 38.8±3.3 | 38.4±3.5 | 0.207 |
| E/e’ | 10.3±2.2 | 10.2±2.3 | 9.8±2.3 | 0.101 |

Data are expressed as mean ± SD, or n (%).

BMI: body mass index; MI: myocardial infarction; PCI: percutaneous coronary intervention; CABG: coronary artery bypass graft; COPD: chronic obstructive pulmonary disease; SBP: systolic blood pressure; DBP: diastolic blood pressure; eGFR: estimated glomerular filtration rate; BNP: B-type natriuretic peptide; HDL-C: high‐density lipoprotein cholesterol; LDL-C: low‐density lipoprotein cholesterol; ACEI/ARB: angiotensin-converting enzyme inhibitor/angiotensin II receptor blocker; CCB: calcium channel blocker; CAD: coronary artery disease; SVD: single vessel disease; DVD: double vessel disease; TVD: triple vessel disease; ACS: acute coronary syndrome; LVEF: left ventricular ejection fraction; LAD: left atrium diameter; E/e’: mitral Doppler early velocity/mitral annular early velocity.
