# Supplementary material for: Incident Heart Failure in Patients With Coronary Artery Disease Undergoing Percutaneous Coronary Intervention
Source: Front Cardiovasc Med. 2021 Oct 4;8:727727. doi: 10.3389/fcvm.2021.727727 (PMC8520925; doi:10.3389/fcvm.2021.727727)
Supplement: Supplementary file 2 [file Table_2.docx]

**Table S2 Multivariate analysis showing predictors of new-onset HFrEF**

|  | SHR | 95% CI | P value |
| --- | --- | --- | --- |
| age | 0.986 | 0.973-1.006 | 0.646 |
| gender (male) | 0.869 | 0.620-1.219 | 0.416 |
| BNP | 2.331 | 1.872-2.902 | <0.001 |
| eGFR | 0.997 | 0.984-1.010 | 0.689 |
| previous MI | 1.694 | 1.093-2.626 | 0.018 |
| AF | 1.642 | 0.788-3.423 | 0.186 |
| hypertension | 0.974 | 0.694-1.366 | 0.878 |
| diabetes | 0.736 | 0.540-1.328 | 0.839 |
| ACS | 0.946 | 0.685-1.306 | 0.735 |
| ACEI/ARB | 0.736 | 0.540-0.986 | 0.042 |
| beta-blocker | 0.957 | 0.704-1.301 | 0.778 |
| multivessel CAD | 1.114 | 0.910-1.314 | 0.295 |
| LVEF | 0.966 | 0.939-0.994 | 0.017 |
| LAD | 1.033 | 0.993-1.075 | 0.109 |
| E/e’ | 1.099 | 1.035-1.166 | <0.001 |

BNP: B-type natriuretic peptide; eGFR: estimated glomerular filtration rate; MI: myocardial infarction; AF: atrial fibrillation; ACS: acute coronary syndrome; ACEI/ARB: angiotensin-converting enzyme inhibitor/angiotensin II receptor blocker; CAD: coronary artery disease; LVEF: left ventricular ejection fraction; LAD: left atrium diameter; E/e’: mitral Doppler early velocity/mitral annular early velocity.
