# Supplementary material for: Incident Heart Failure in Patients With Coronary Artery Disease Undergoing Percutaneous Coronary Intervention
Source: Front Cardiovasc Med. 2021 Oct 4;8:727727. doi: 10.3389/fcvm.2021.727727 (PMC8520925; doi:10.3389/fcvm.2021.727727)
Supplement: Supplementary file 3 [file Table_3.docx]

**Table S3 Multivariate analysis showing predictors of new-onset HfmrEF**

|  | SHR | 95% CI | P value |
| --- | --- | --- | --- |
| age | 0.979 | 0.961-1.008 | 0.633 |
| gender (male) | 0.809 | 0.529-1.236 | 0.326 |
| BNP | 2.810 | 2.137-3.694 | <0.001 |
| eGFR | 0.999 | 0.981-1.018 | 0.929 |
| previous MI | 1.078 | 0.586-1.985 | 0.808 |
| AF | 2.605 | 1.212-5.599 | 0.014 |
| hypertension | 1.013 | 0.657-1.562 | 0.953 |
| diabetes | 1.063 | 0.711-1.592 | 0.764 |
| ACS | 1.348 | 0.896-2.028 | 0.152 |
| ACEI/ARB | 0.736 | 0.498-0.925 | 0.035 |
| beta-blocker | 0.881 | 0.601-1.292 | 0.517 |
| multivessel CAD | 1.062 | 0.828-1.361 | 0.635 |
| LVEF | 0.990 | 0.952-1.030 | 0.611 |
| LAD | 1.019 | 0.972-1.067 | 0.436 |
| E/e’ | 1.092 | 1.003-1.187 | 0.042 |

BNP: B-type natriuretic peptide; eGFR: estimated glomerular filtration rate; MI: myocardial infarction; AF: atrial fibrillation; ACS: acute coronary syndrome; ACEI/ARB: angiotensin-converting enzyme inhibitor/angiotensin II receptor blocker; CAD: coronary artery disease; LVEF: left ventricular ejection fraction; LAD: left atrium diameter; E/e’: mitral Doppler early velocity/mitral annular early velocity.

| **Variables in the Equation** | | | | | | | | |
| --- | --- | --- | --- | --- | --- | --- | --- | --- |
|  | B | SE | Wald | df | Sig. | Exp(B) | 95.0% CI for Exp(B) | |
|  |  |  |  |  |  |  | Lower | Upper |
| age | -.009 | .009 | .901 | 1 | .343 | .991 | .974 | 1.009 |
| gender | -.316 | .205 | 2.379 | 1 | .123 | .729 | .488 | 1.089 |
| BNP2 | 1.187 | .156 | 58.026 | 1 | .000 | 3.278 | 2.415 | 4.450 |
| egfr | -.006 | .009 | .437 | 1 | .509 | .994 | .978 | 1.011 |
| previousmi | .567 | .302 | 3.532 | 1 | .060 | 1.764 | .976 | 3.187 |
| af | 1.110 | .397 | 7.809 | 1 | .005 | 3.033 | 1.393 | 6.605 |
| hypertension | -.072 | .224 | .102 | 1 | .749 | .931 | .601 | 1.443 |
| diabetes | .004 | .206 | .000 | 1 | .984 | 1.004 | .670 | 1.504 |
| acs | .315 | .200 | 2.477 | 1 | .116 | 1.370 | .926 | 2.028 |
| aceiarb | -.282 | .198 | 2.026 | 1 | .155 | .754 | .511 | 1.112 |
| betablocker | -.248 | .200 | 1.541 | 1 | .214 | .780 | .527 | 1.155 |
| vd2 | .493 | .241 | 4.194 | 1 | .041 | 1.638 | 1.021 | 2.626 |
| lvef | -.015 | .019 | .620 | 1 | .431 | .985 | .948 | 1.023 |
| lad | .016 | .026 | .383 | 1 | .536 | 1.016 | .966 | 1.068 |
| ee | .016 | .041 | .158 | 1 | .691 | 1.016 | .938 | 1.102 |
