# Supplementary material for: Incident Heart Failure in Patients With Coronary Artery Disease Undergoing Percutaneous Coronary Intervention
Source: Front Cardiovasc Med. 2021 Oct 4;8:727727. doi: 10.3389/fcvm.2021.727727 (PMC8520925; doi:10.3389/fcvm.2021.727727)
Supplement: Supplementary file 4 [file Table_4.docx]

**Table S4 Multivariate analysis showing predictors of new-onset HFpEF**

|  | SHR | 95% CI | P value |
| --- | --- | --- | --- |
| age | 1.002 | 0.988-1.017 | 0.711 |
| gender (male) | 0.725 | 0.531-0.990 | 0.043 |
| BNP | 1.183 | 0.969-1.445 | 0.100 |
| eGFR | 0.981 | 0.968-0.994 | 0.006 |
| previous MI | 1.088 | 0.683-0.732 | 0.631 |
| AF | 4.854 | 3.096-7.610 | <0.001 |
| hypertension | 1.399 | 0.996-1.966 | 0.053 |
| diabetes | 1.400 | 1.052-1.861 | 0.021 |
| ACS | 1.346 | 1.018-1.780 | 0.037 |
| ACEI/ARB | 0.826 | 0.697-0.986 | 0.036 |
| beta-blocker | 1.234 | 0.924-1.649 | 0.155 |
| multivessel CAD | 1.039 | 0.872-1.238 | 0.668 |
| LVEF | 0.999 | 0.970-1.028 | 0.921 |
| LAD | 1.010 | 0.973-1.050 | 0.597 |
| E/e’ | 1.045 | 0.992-1.108 | 0.078 |

BNP: B-type natriuretic peptide; eGFR: estimated glomerular filtration rate; MI: myocardial infarction; AF: atrial fibrillation; ACS: acute coronary syndrome; ACEI/ARB: angiotensin-converting enzyme inhibitor/angiotensin II receptor blocker; CAD: coronary artery disease; LVEF: left ventricular ejection fraction; LAD: left atrium diameter; E/e’: mitral Doppler early velocity/mitral annular early velocity.
